# Extracellular NGFR Spacers Allow Efficient Tracking and Enrichment of Fully Functional CAR-T Cells Co-Expressing a Suicide Gene

**DOI:** 10.3389/fimmu.2018.00507

**Published:** 2018-03-21

**Authors:** Monica Casucci, Laura Falcone, Barbara Camisa, Margherita Norelli, Simona Porcellini, Anna Stornaiuolo, Fabio Ciceri, Catia Traversari, Claudio Bordignon, Chiara Bonini, Attilio Bondanza

**Affiliations:** ^1^Innovative Immunotherapies Unit, Division of Immunology, Transplantation and Infectious Diseases, San Raffaele Hospital Scientific Institute, Milano, Italy; ^2^Molmed Spa, Milano, Italy; ^3^Vita-Salute San Raffaele University, Milano, Italy; ^4^Hematology and Bone Marrow Transplantation Unit, San Raffaele Hospital Scientific Institute, Milano, Italy; ^5^Experimental Hematology Unit, Division of Immunology, Transplantation and Infectious Diseases, San Raffaele Hospital Scientific Institute, Milano, Italy

**Keywords:** CAR-T cells, CAR spacer, cell sorting, good manufacturing procedures-manufacturing, antitumor efficacy, suicide gene

## Abstract

Chimeric antigen receptor (CAR)-T cell immunotherapy is at the forefront of innovative cancer therapeutics. However, lack of standardization of cellular products within the same clinical trial and lack of harmonization between different trials have hindered the clear identification of efficacy and safety determinants that should be unveiled in order to advance the field. With the aim of facilitating the isolation and *in vivo* tracking of CAR-T cells, we here propose the inclusion within the CAR molecule of a novel extracellular spacer based on the low-affinity nerve-growth-factor receptor (NGFR). We screened four different spacer designs using as target antigen the CD44 isoform variant 6 (CD44v6). We successfully generated NGFR-spaced CD44v6 CAR-T cells that could be efficiently enriched with clinical-grade immuno-magnetic beads without negative consequences on subsequent expansion, immuno-phenotype, *in vitro* antitumor reactivity, and conditional ablation when co-expressing a suicide gene. Most importantly, these cells could be tracked with anti-NGFR monoclonal antibodies in NSG mice, where they expanded, persisted, and exerted potent antitumor effects against both high leukemia and myeloma burdens. Similar results were obtained with NGFR-enriched CAR-T cells specific for CD19 or CEA, suggesting the universality of this strategy. In conclusion, we have demonstrated that the incorporation of the NGFR marker gene within the CAR sequence allows for a single molecule to simultaneously work as a therapeutic and selection/tracking gene. Looking ahead, NGFR spacer enrichment might allow good manufacturing procedures-manufacturing of standardized CAR-T cell products with high therapeutic potential, which could be harmonized in different clinical trials and used in combination with a suicide gene for future application in the allogeneic setting.

## Introduction

Chimeric antigen receptors (CARs) are synthetic biology molecules constructed by fusing an extracellular antigen-binding moiety, commonly the single-chain variable fragment (scFv) of a tumor-reactive monoclonal antibody (mAb), with intracellular activatory units, usually the CD3 zeta chain, coupled with costimulatory endodomains from either CD28 or 41BB (1). Over the last 5 years, different US Institutions have shown impressive antitumor effects after infusing CD19 CAR-T cells in patients with refractory B-cell malignancies, including chronic lymphocytic leukemia (2, 3), B-cell acute lymphoblastic leukemia (4–7) and non-Hodgkin lymphomas (2, 8). More recently, the approval of the first CAR-T cell therapy to treat pediatric and young adult relapsed/refractory B-cell ALL has landmarked the beginnings of what will likely be a new era in cancer therapeutics.

Accumulating clinical data suggest that primary expansion and long-term *in vivo* persistence of CAR-T cells are main determinants of the final therapeutic outcome. These properties are seemingly influenced by both CAR-T cell and host-specific factors. For instance, CAR designs including CD28 (9) and 41BB (10) costimulatory endodomains, as well as the frequencies of stem (T_SCM_) and central memory (T_CM_) T cells in the final product (11), have both been shown to substantially contribute to a long-lived phenotype. On the other hand, patient pre-conditioning is recognized to promote CAR-T cell engraftment (7, 12), while contrariwise residual host immunity may cause their humoral and/or T-cell mediated rejection, especially if murine scFv sequences are used (7, 13, 14). Related to this, while using human scFv may drastically reduce the immunogenicity of synthetic CARs, prediction algorithms may be exploited to evaluate the potential of fusion sites between human components to provide immunogenic epitopes for T-cell immune responses, allowing their preventive modification (15). As CAR-T cells are entering the commercial phase, investigators, regulators, and industrial stakeholders are dedicating increasing attention to the pharmaceutical aspects of this revolutionary type of treatment, including rationalization of good manufacturing procedures and in-depth analysis of toxicology, pharmacokinetics, and pharmacodynamics (16). These continuing efforts clearly require new, easy and informative methods for tracking and characterizing transgene-expressing and, therefore, pharmacologically active T cells, both in the final CAR-T cell product before infusion and, later, in treated patients.

Currently available tracking methods rely on qPCR (4, 5, 17) or on antibodies specific for either the CAR molecule itself (11, 18) or a separate marker gene (7, 8, 19). Compared with PCR, antibody-based methods have the advantage of enabling not only the *in vivo* tracking of CAR-T cells, but also the characterization, at a single-cell level, of their differentiation, activation, and exhaustion statuses. In addition, they offer the unique possibility to enrich CAR-T cells before infusion, allowing the design of more standardized CAR-T cell therapies. In foresight, this possibility might crucially facilitate the translation of CAR-T cells to the allogeneic setting, where coexpressing a suicide gene would necessarily require an enrichment step to remove unmodified alloreactive cells (20). Unfortunately, the antibody-based methods for CAR-T cell marking developed so far have some limitations, especially in light of their potential use as universal enrichment tools. For instance, anti-idiotypic mAbs already used for CD19 CARs (18) would need to be developed for each single specificity and, if used for enrichment, are expected to unduly activate CAR-T cells during *ex vivo* manipulation. On the other hand, separate immuno-marker genes (7, 8, 19) reflect CAR expression only indirectly and may saturate the cargo capacity of currently available viral vectors, abating transduction efficiency, especially in the case of multi-cistronic cassettes (CAR, immune-marker and suicide gene).

A promising alternative to these approaches is the inclusion of an immuno-marker sequence within the extracellular portion of the CAR molecule itself. In this study, we designed an innovative CAR spacer based on extracellular domains from the low-affinity nerve-growth-factor receptor (NGFR), a marker gene already used in the clinic for the selection/tracking of transduced T cells. We then validated the antitumor efficacy of NGFR-enriched CAR-T cells specific for the CD44 isoform variant 6 (CD44v6), CD19, and CEA in clinically relevant *in vivo* xenograft mouse models. Additionally, we engineered T cells with a clinical-grade bi-cistronic retroviral vector encoding for the NGFR-spaced CD44v6 CAR and the thymidine kinase (TK) suicide gene and proved efficient sorting with clinical-grade reagents, potent antitumor efficacy and optimal suicidability upon exposure to Ganciclovir. This NGFR-spaced CD44v6 CAR T-cell product is currently at late stage of process development and these efforts have recently gained *momentum* by the EC through dedicated H2020 funding to support phase I/IIa clinical trial in patients with relapsed/refractory acute myeloid leukemia (AML) and multiple myeloma (MM).

## Materials and Methods

### Construct Generation

We used the low-affinity NGFR gene as reference (P08138, TNR16_HUMAN). The NGFR wild-type long (NWL) construct contains the four TNFR cysteine-rich domains and the serine/threonine-rich stalk. The NGFR wild-type short (NWS) construct contains only the four TNFR cysteine-rich domains. The NGFR mutated long (NML) construct contains the four TNFR cysteine-rich domains and the stalk, but the fourth domain was largely deleted to avoid NGF signaling (21). The NGFR mutated short (NMS) contains only the four TNFR cysteine-rich domains, with the mutated version of the fourth domain. NGFR spacers were synthetized by GeneArt (ThermoFisher) and cloned into an original CAR incorporating an IgG1-derived CH2CH3 spacer, the CD28 transmembrane and costimulatory domains and the CD3 zeta chain (9). The same procedure was applied to generate NGFR isoform-spaced CARs specific for CD19 and the carcinoembryonic antigen. All constructs were expressed into SFG mono-cistronic retroviral vectors (22) under the direct control of viral LTR. In a specific set of experiments, NWL and NMS-isoform-spaced CD44v6 CARs were expressed into a clinical-grade bi-cistronic retroviral vector in combination with the TK suicide gene (SFCMM-3) (20). In these constructs, CAR genes were placed under control of the internal SV40 promoter in place of the ΔNGFR marker gene while TK was maintained under the control of viral LTR (see Figure 7B).

### Flow Cytometry

We used fluorochrome-conjugated mouse mAbs specific for human CD3, CD4, CD8, CD14, CD16, CD19, CD32, CD33, CD38, CD44v6, CD45, CD45RA, CD62L, CD64, PD1, HLA-DR, and NGFR (CD271, clone C40-1457, BD Biosciences; clone ME20.4, Miltenyi Biotec), mouse polyclonal antibodies specific for human IgG1-CH2CH3 (Jackson Laboratories, for staining CH2CH3-spaced CAR T cells), rat mAb specific for mouse CD45 (Ly5.1) and recombinant Protein L (ThermoFisher Scientific, for staining CAR’s scFv). Died cells were identified by positive staining with DAPI (BD Biosciences). Cells were acquired with a FACSCanto II apparatus (BD Biosciences). Data were analyzed with the FlowJo software (Tree Star, Inc.) and relative fluorescence intensity was calculated as follows: mean fluorescence intensity (MFI) of the sample stained with the mAb of interest/MFI of the sample stained with an isotype-matched control.

### Transduction and Culture Conditions

Buffy coats from healthy blood donors were obtained after written informed consent and IRB approval. T cells were stimulated with CD3/CD28-beads (CTS Dynabeads CD3/CD28, ThermoFisher), RV-transduced with two rounds of spinoculation, and cultured in RPMI 1640 (Gibco-Brl), 10% FBS (EuroClone) with IL-7/IL-15 (5 ng/ml, PeproTech). After 6 days, beads were removed and, after additional 3–7 days, CAR-T cells were stained with the PE-conjugated anti-NGFR mAb C40-1457 and enriched with anti-PE immune-magnetic beads (Miltenyi Biotech). In a specific set of experiments (Figure 7), T cells were stimulated with CD3/CD28-beads and RV-transduced into a RetroNectin coated bag in X-Vivo 15, 3% plasma with IL-7/IL-15 (100 and 200 U/ml, respectively). The day after, beads were removed and, after additional 3 days, NWL-isoform-spaced CAR-T cells were enriched with clinical-grade anti-NGFR immune-magnetic beads (CD271 Microbeads, Miltenyi Biotec), while NMS-isoform-spaced CAR-T cells were enriched with the two-step procedure described above. CAR-T cell expansion is expressed as fold increase over numbers before enrichment.

### *In Vitro* Functional Assays

Immuno-enriched CAR-T cells were cocultured at different E:T ratios with the following tumor-cell lines: MM.1S myeloma cells, THP-1 and HL-60 myeloid leukemic cells, BV-173 lymphoid leukemic cells, BxPC-3 pancreatic tumor cells, ALL-CM leukemic cells [kindly provided by Fred Falkenburg, Leiden University Medical Center (23)], primary AML blasts and MM plasma cells (from our Institutional Biobank after IRB approval). T cells transduced with an irrelevant CAR were always used as control (CTRL, GD2, or CD19-specific as indicated). Twenty-four-hour supernatants were collected and subsequently analyzed by FACS using the LEGENDplex cytokine immunoassay (BioLegend). Four-day cocultures were analyzed by FACS using Flow-Count Fluorospheres (BeckmanCoulter). The elimination index was calculated as follows: 1 − (number of residual target cells in presence of target antigen-specific CAR-T cells/number of residual target cells in presence of CTRL CAR-T cells). In cell proliferation assays, immune-enriched CAR-T cells were labeled with 0.2 µM carboxy fluorescein succinimidyl ester (CFSE, Invitrogen), washed, and stimulated with irradiated (10,000 rad) tumor cells at 1:5 E:S ratio. After 6 days, the cells were analyzed by FACS and proliferation expressed as the percentage of CFSE-diluting cells. In a specific set of experiments (Figure 7), the efficacy of the suicide gene machinery was evaluated by exposing PHA-activated (2 µg/ml, Sigma-Aldrich) CAR T cells to increasing concentrations of GCV. Cell viability was analyzed 5 days after by trypan blue exclusion and calculated as follows: number of living cells in GCV-treated samples/number of living cells in untreated samples × 100. T cells transduced with the original SCFMM-3 construct carrying TK and ΔNGFR as a separate selection marker were used as comparison.

### Studies in Xeno-Engrafted NSG Mice

All mouse experiments were approved by the Institutional Animal Care and Use Committee (IACUC #468) of San Raffaele University Hospital and Scientific Institute and by the Italian Governmental Health Institute (Rome, IT). 6- to 8-week-old NSG mice were obtained from the Jackson Laboratories. In the high-tumor burden settings, mice were injected intravenously (i.v.) with tumor cells transduced with a lentiviral vector encoding for the secreted luciferase Lucia (24) (THP-1-luc cells, 1.5 × 10^6^; MM.1S-luc cells, 2 × 10^6^) and, after 14 (THP-1) or 28 (MM.1S) days, treated with immuno-enriched CAR-T cells (5 × 10^6^) by i.v. infusion. Tumor progression was monitored weekly by bioluminescence using the QUANTI-Luc detection reagent (InvivoGen) and expressed as relative light units (RLUs), according to the manufacturer instructions. Circulating human T-cell counts were measured weekly by FACS using Flow-Count Fluorospheres (BeckmanCoulter). Mice were sacrificed when RLUs were >4 × 10^7^ for THP-1-luc cells or >4 × 10^5^ for MM.1S-luc cells, or when manifesting clinical signs of suffering. *In vivo* cytokine concentrations were measured by FACS using the LEGENDplex immunoassay (BioLegend). In a specific set of experiments (Figure 7), mice were injected with THP-1 cells and, after 14 days, treated with immuno-enriched CAR-T cells. At day 40, mice were sacrificed and weights of THP1-infiltrated livers were analyzed. In the minimal residual-disease settings, mice were injected i.v. with either THP-1 or ALL-CM cells (5 × 10^6^) and, after 3 days, treated with immune-enriched CAR-T cells (5 × 10^6^) by i.v. infusion. Circulating human T and leukemic-cell counts were measured weekly by FACS using Flow-Count Fluorospheres (BeckmanCoulter). At sacrifice (THP-1, day 35; ALL-CM, day 45), liver, bone marrow, and spleen were analyzed for the presence of residual leukemic cells.

### Statistical Analysis

Statistical analyzes were conducted using Prism Software 5.0 (GraphPad). We analyzed the datasets with paired or unpaired Student’s *t*-test, one-way ANOVA or the Log-rank Mantel–Cox tests. Differences with a *P* value < 0.05 were considered statistically significant.

## Results

### NGFR-Spaced CAR-T Cells Can Be Tracked and Enriched With Anti-NGFR Reagents

**Figure 1:**
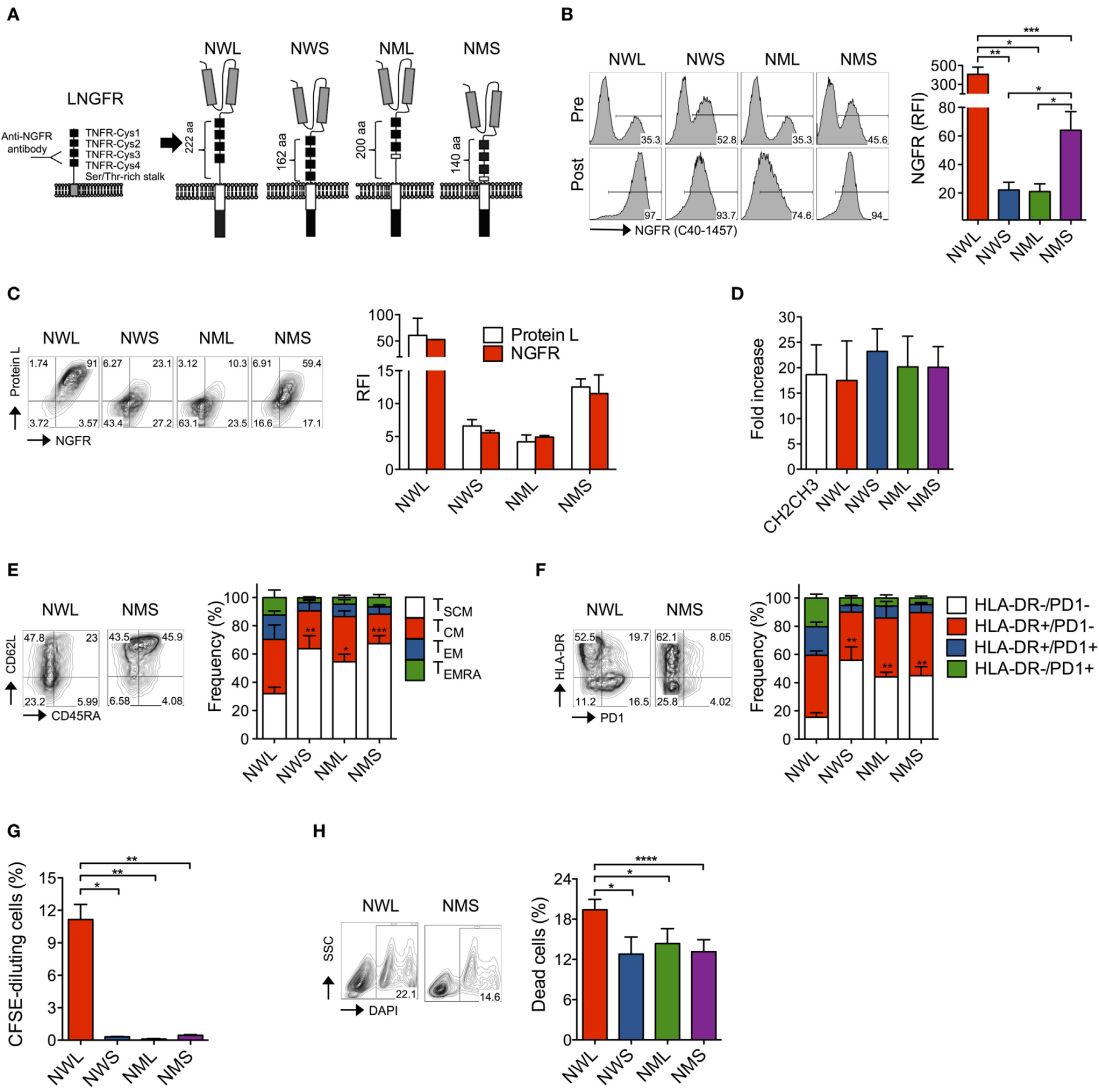
Generation of nerve-growth-factor receptor (NGFR)-spaced CD44v6 chimeric antigen receptor (CAR)-T cells. **(A)** Structure of the low-affinity NGFR extracellular region and of the four NGFR isoform-spaced CD44v6 CAR constructs (NWL, NGFR wild-type long; NWS, NGFR wild-type short; NML, NGFR mutated long; NMS, NGFR mutated short). Gray boxes: single-chain variable fragment (scFv); white box: CD28 costimulatory/transmembrane endodomain; black box: CD3 zeta chain. Primary T cells from healthy donors were stimulated with CD3/CD28-beads, transduced with RVs encoding for CD44v6 CARs spaced with NWL, NWS, NML, or NMS, enriched with immuno-magnetic beads (see Materials and Methods) and cultured with IL-7/IL-15. **(B)** Left: FACS plots of CAR-T cells from a representative donor (of *n* = 8) before (pre) and after (post) enrichment. Right: NGFR expression on CAR-T cells showed as relative fluorescence intensities (RFIs) over isotype-matched controls (means ± SEM from *n* = 8 donors). Results from a paired *t*-test are indicated when statistically significant (*P ≤ 0.05; **P ≤ 0.01; ***P ≤ 0.001). **(C)** Left: FACS plots of resting CAR-T cells from a representative donor stained with NGFR (*x* axes) and Protein L (*y* axes). Right: NGFR and Protein L expression on CAR-T cells showed as RFIs over isotype-matched controls (means ± SEM from *n* = 3 donors). **(D)** Expansion of NGFR isoform- or CH2CH3-spaced CD44v6 CAR-T cells expressed as fold increase at day 16–20 after bead stimulation (see Materials and Methods, means ± SEM from *n* = 5 donors). **(E)** Memory phenotypes of NGFR isoform-enriched CD44v6 CAR-T cells analyzed at day 16–20 after bead stimulation. Left: FACS plots from a representative donor. Right: data obtained from *n* = 7 donors (means ± SEM). Results from a paired t-test on TSCM are indicated when statistically significant (*P ≤ 0.05; **P ≤ 0.01; ***P ≤ 0.001). TSCM, CD45RA+/CD95+/CD62L+ stem memory T cells; TCM, CD45RA−/CD95+/CD62L+ central memory T cells; TEM, CD45RA−/CD95+/CD62L− effector memory T cells; TEMRA, CD45RA+/CD95+/CD62L− effector memory RA T cells. **(F)** Exhaustion/activation phenotypes of NGFR isoform-enriched CD44v6 CAR-T cells analyzed at day 16–20 after bead stimulation. Left: FACS plots from a representative donor. Right: data obtained from *n* = 3 donors (means ± SEM). Results from a paired *t*-test on HLA-DR−/PD1− cells are indicated when statistically significant (**P ≤ 0.01). **(G)** NGFR isoform-enriched CD44v6 CAR-T cells were labeled with CFSE and analyzed by FACS after 6-day culture in the absence of cognate antigen stimulation. Proliferation was expressed as percentages of CFSE-diluting cells (means ± SEM from *n* = 3 donors). Results from a paired *t*-test are indicated when statistically significant (*P ≤ 0.05; **P ≤ 0.01). **(H)** Mortality rate of NGFR isoform-enriched CD44v6 CAR-T cells expressed as percentages of dead DAPI + cells at day 16–20 after bead stimulation. Left: FACS plots from a representative donor. Right: data obtained from *n* = 7 donors (means ± SEM). Results from a paired *t*-test are indicated when statistically significant (*P ≤ 0.05; ***P ≤ 0.001).

We recently developed a CAR specific for CD44v6 for targeting AML and MM, as well as multiple epithelial tumors (25). However, our original CAR construct was equipped with an IgG1-CH2CH3 spacer (CH2CH3) that could reduce its antitumor activity *in vivo* due to undesired interaction with Fc receptors (FcR)-bearing myeloid cells, as previously reported (26–30). A truncated version of the low-affinity NGFR lacking intracellular signaling components is currently used in the clinic for gene marking and enrichment of engineered T cells (20, 31). Aiming at developing a construct that could simultaneously enable the enrichment and tracking of fully functional CD44v6 CAR-T cells, we replaced the CH2CH3 spacer with the extracellular domain from the NGFR. In particular, we generated four isoforms of the NGFR spacer, namely the wild-type long (NWL), the wild-type short (NWS), the mutated long (NML), and the mutated short (NMS), differing in length and inclusion or not of a specific deletion abrogating NGF signaling (21) (Figure 1A; see Materials and Methods).

We cloned the different NGFR isoform-spaced CD44v6 CAR constructs into retroviral vectors (22, 25) under the control of viral LTR driving strong transgene expression and transduced primary T cells after activation with CD3/CD28-beads and IL-7/IL-15, according to a protocol that enriches for stem (T_SCM_) and central memory (T_CM_) T cells (32–34). After transduction, all isoforms could be identified on the cell surface using the anti-NGFR mAb C40-1457 and could be enriched with immuno-magnetic beads (Figure 1B). Interestingly, different NGFR staining intensities between isoforms (NWL > NMS ≫ NWS/NML) were paralleled by staining with Protein L, a reagent that specifically binds to scFv (35) (Figure 1C), suggesting that different surface CAR stability, rather than epitope accessibility, was responsible for these differences.

After enrichment, all NGFR isoform-spaced CAR-T cells expanded similarly to those enriched through CH2CH3 (Figure 1D). Interestingly, however, NWL-enriched CAR-T cells displayed a lower proportion of T_SCM_ cells compared to NWS-, NML-, or NMS-enriched CAR-T cells (Figure 1E) and a higher proportion of T cells expressing HLA-DR and PD1, alone or in combination (Figure 1F), suggesting that the NWL-isoform might have induced tonic signaling, as already reported for CH2CH3 spacers (29). As a confirmation, even in the absence of cognate antigen, NWL-enriched CAR-T cells displayed a basal level of proliferation (Figure 1G), and a higher apoptotic rate (Figure 1H) compared to CAR-T cells enriched through other NGFR isoform spacers.

### NGFR-Enriched CD44v6 CAR-T Cells Are Fully Functional *In Vitro*

**Figure 2:**
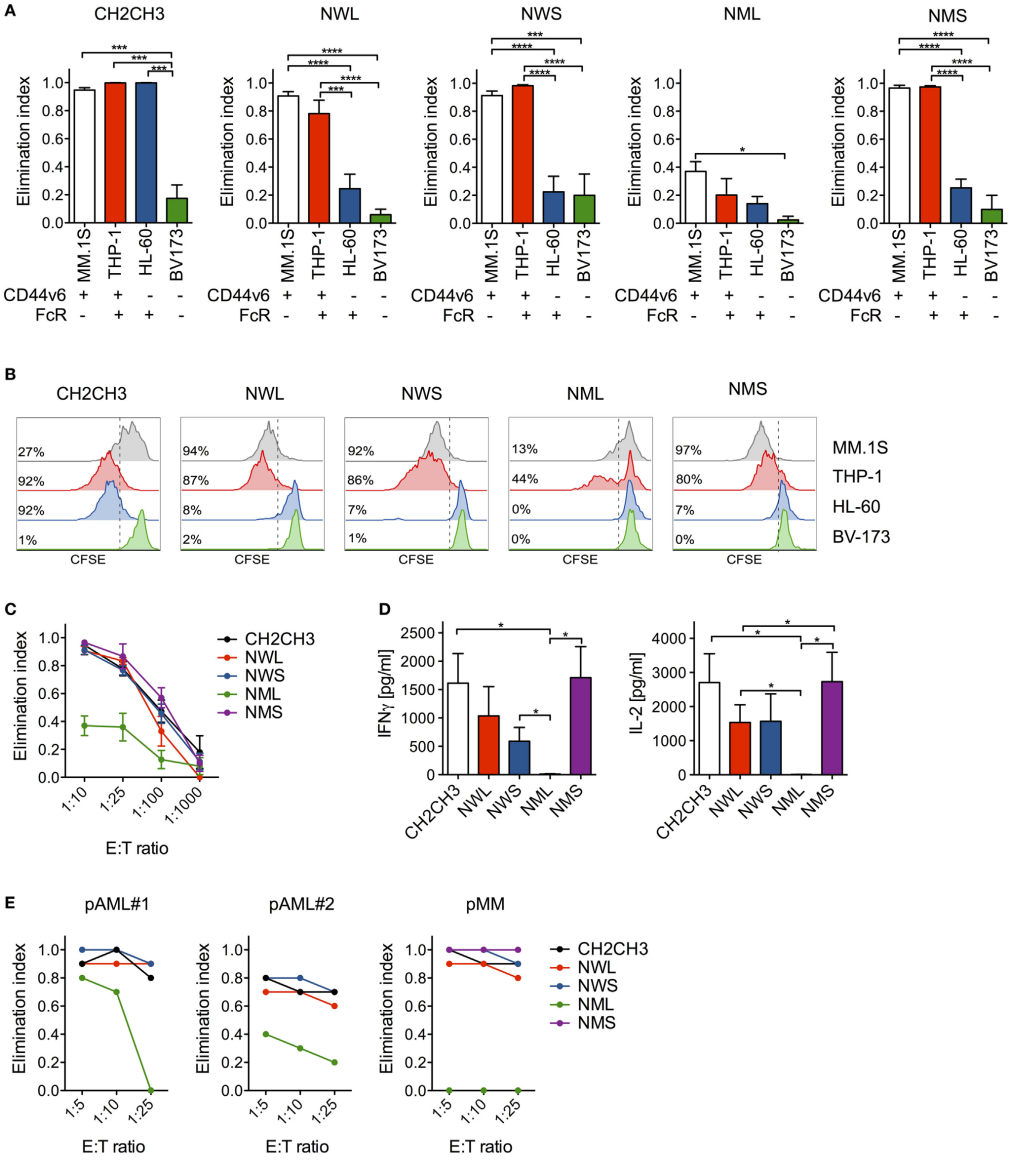
*In vitro* antitumor activity by nerve-growth-factor receptor (NGFR)-enriched CD44v6 chimeric antigen receptor (CAR)-T cells. **(A)** CD44v6 CAR-T cells enriched through either CH2CH3, NGFR wild-type long (NWL), NGFR wild-type short (NWS), NGFR mutated long (NML), or NGFR mutated short (NMS) were cocultured at 1:10 E:T (Effector:Target) ratio with tumor cell lines differentially expressing CD44v6 and/or Fc receptors (FcRs) (tumor cells were considered FcR+ if expressing either CD16, CD32, CD64, or any of their combination). After 4 days, the killing of tumor cells by CAR-T cells were analyzed by FACS and expressed as elimination indexes (see Materials and Methods, means ± SEM from *n* = 5 donors). Results from a one-way ANOVA test are indicated when statistically significant (*P ≤ 0.05; ***P ≤ 0.001; ****P ≤ 0.0001). **(B)** CH2CH3- and NGFR isoform-enriched CD44v6 CAR-T cells were labeled with CFSE and stimulated at 1:5 E:S (Effector:Stimulator) ratio with the same tumor cell lines. After 6 days, CAR-T cell proliferation was analyzed by FACS and expressed as percentages (inserts) of CFSE-diluting cells. FACS plots of CAR-T cells from a representative donor of *n* = 3 are depicted. **(C)** CH2CH3- and NGFR isoform-enriched CD44v6 CAR-T cells were cocultured at limiting E:T ratios with multiple myeloma (MM).1S myeloma cells. After 4 days, the killing of MM.1S cells by CAR-T cells were analyzed by FACS and expressed as elimination indexes (see Materials and Methods, means ± SEM from *n* = 5 donors). **(D)** Concentrations of IFN-γ (left) and IL-2 (right) measured in 24 h coculture supernatants of CH2CH3- and NGFR isoform-enriched CD44v6 CAR-T cells (means ± SEM from *n* = 4 donors). Results from a one-way ANOVA test are shown when statistically significant (*P ≤ 0.05). **(E)** CH2CH3- and NGFR isoform-enriched CD44v6 CAR-T cells from one donor were cocultured at different E:T ratios with CD44v6+ primary malignant plasma cells (pMM) and primary leukemic blasts (pAML#1, pAML#2) from patients. After 4 days, residual tumor cells were counted and analyzed by FACS. The elimination indexes by CAR-T cells are shown against each primary target-cell population.

To verify the functionality of NGFR-enriched CD44v6 CAR-T cells, we tested them in coculture experiments with tumor cells expressing or not CD44v6. Similarly to cells enriched through the CH2CH3 spacer, NWL-, NWS-, and NMS-, but not NML-enriched CAR-T cells killed CD44v6+ THP-1 myeloid leukemia cells and MM.1S myeloma cells, while spared CD44v6− BV-173 lymphoid leukemia cells (Figure 2A). On the contrary, they expectedly failed to kill CD44v6−/FcR+ HL-60 myeloid leukemia cells, indicating efficient abrogation of FcR recognition. Accordingly, NWL-, NWS-, and NMS-, but not NML-enriched effectors secondarily proliferated upon coculture with CD44v6+, but not with CD44v6− tumor cells (Figure 2B).

To exclude that replacing the CH2CH3 spacer with NGFR spacers might have reduced antitumor reactivity *in vitro*, we challenged NGFR-enriched CD44v6 CAR-T cells against CD44v6+/FcR− MM.1S cells at limiting E:T ratios. Importantly, NWL-, NWS-, and NMS-, but not NML-enriched CAR-T cells, were as cytotoxic as those enriched through CH2CH3 (Figure 2C). The maintenance of potent antitumor reactivity was confirmed by cytokine production in response to MM.1S cells (Figure 2D) and, most importantly, by efficient killing of both primary AML blasts and malignant plasma cells (Figure 2E).

**Figure 3:**
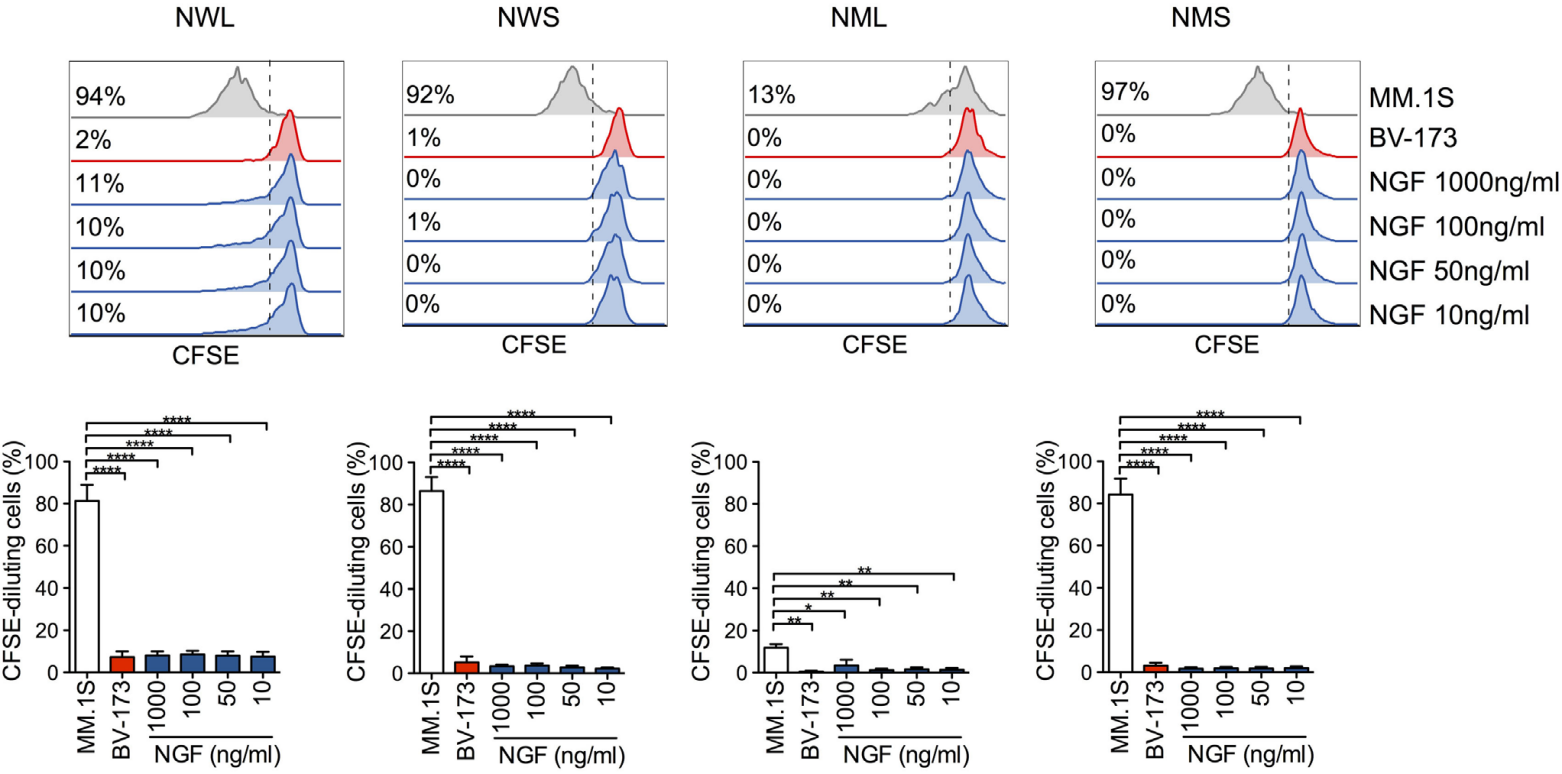
Lack of activation of nerve-growth-factor receptor (NGFR)-enriched CD44v6 chimeric antigen receptor (CAR)-T cells in response to NGF. NGFR isoform-enriched CD44v6 CAR-T cells were labeled with CFSE and exposed to increasing concentrations of soluble NGF. CD44v6+ MM.1S and CD44v6− BV-173 cells were used as positive and negative stimuli, respectively. After 6 days, CAR-T cell proliferation was analyzed by FACS and expressed as percentages (inserts) of CFSE-diluting cells. Upper panel: FACS plots of CAR-T cells from a representative donor. Lower panel: percentages of CFSE-diluting cells from *n* = 3 donors (means ± SEM). Results from a one-way ANOVA test are shown when statistically significant (*P ≤ 0.05; **P ≤ 0.01; ****P ≤ 0.0001).

Importantly, while exposing NGFR-enriched CD44v6 CAR-T cells to CD44v6+ MM.1S cells induced secondary proliferation, adding soluble NGF at concentrations capable of forcing neuronal tumor-cell differentiation had no effect (Figure 3), ruling out a potential proliferative advantage of NGFR-enriched CD44v6 CAR-T cells upon encounter with its natural ligand NGF.

### NGFR-Enriched CD44v6 CAR-T Cells Mediate Potent Therapeutic Effects Against High-Leukemia and Myeloma Burdens in NSG Mice

**Figure 4:**
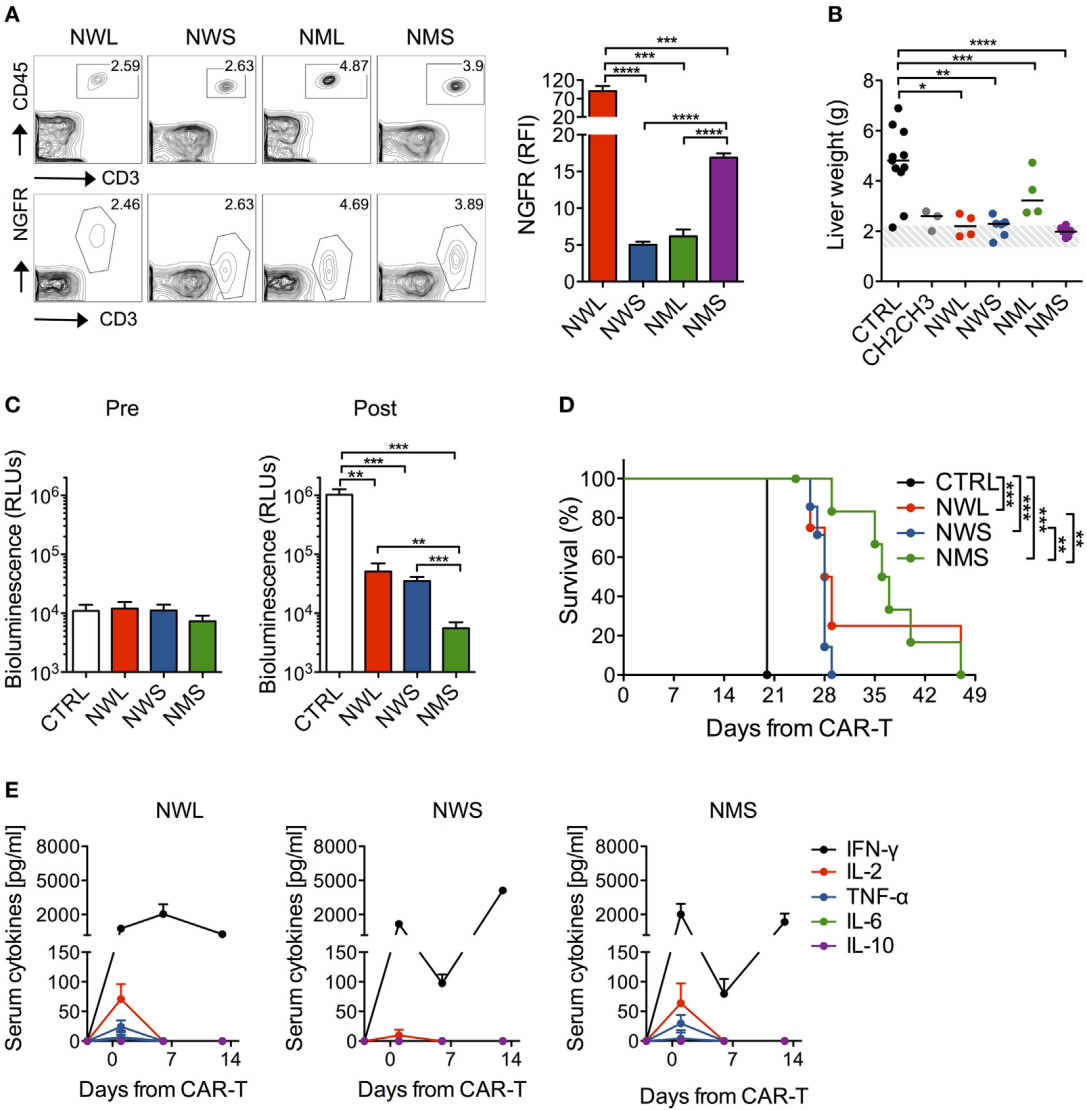
*In vivo* antileukemia activity by nerve-growth-factor receptor (NGFR)-enriched CD44v6 chimeric antigen receptor (CAR)-T cells. NSG mice were xeno-engrafted with THP-1 leukemic cells and, after 3 days, treated with CD44v6 CAR-T cells enriched through either CH2CH3 (*n* = 3), NGFR wild-type long (NWL) (*n* = 4), NGFR wild-type short (NWS) (*n* = 6), NGFR mutated long (NML) (*n* = 4), or NGFR mutated short (NMS) (*n* = 6), or with T cells expressing a control, GD2-specific CAR (CTRL, *n* = 11). **(A)** Left: FACS plots of human circulating T cells (upper panels) and of their NGFR expression (lower panels) 3 days after treatment in a representative mouse per cohort. Right: NGFR expression *in vivo* expressed as relative fluorescence intensities (RFIs) over isotype-matched controls (means ± SEM from the different cohorts). Results from an unpaired *t*-test are indicated when statistically significant (***P ≤ 0.001; ****P ≤ 0.0001). **(B)** Thirty-five days later, mice were sacrificed and their liver weighted. THP-1 cell-infiltrated liver weights are shown for the different cohorts (each symbol represents a single mouse and the median value for each group is reported). The dashed area depicts the range of normal liver weight from age/sex-matched normal NSG mice. Results from a one-way ANOVA test are indicated when statistically significant (*P ≤ 0.05; **P ≤ 0.01; ***P ≤ 0.001; ****P ≤ 0.0001). **(C)** In another experiment, NSG mice were xeno-engrafted with THP-1 cells expressing a secreted luciferase (see Materials and Methods) and, after 14 days (arrow), treated with CD44v6 CAR-T cells enriched through either NWL (*n* = 4), NWS (*n* = 7) or NMS (*n* = 7), or with CTRL, CD19-specific CAR-T cells (*n* = 4). THP-1-luc cell-derived bioluminescence was measured in the peripheral blood (see Methods) the day before (Pre) or 6 days after (Post) CAR-T cell infusion and expressed as relative light units (RLUs, means ± SEM from the different cohorts). Results from an unpaired *t*-test are indicated when statistically significant (**P ≤ 0.01; ***P ≤ 0.001). **(D)** Survival of the different cohorts over time is shown as percentages. Results from Log-rank (Mantel–Cox) tests are indicated when statistically significant (**P ≤ 0.01; ***P ≤ 0.001). **(E)** Systemic levels of human IFN-γ, IL-2, TNF-α, IL-6, and IL-10 measured in the serum of mice before and at different time points after NGFR isoform-enriched CAR-T cell infusion are shown (mean concentrations ± SEM from the different cohorts).

We next sought to validate the antitumor efficacy of NGFR-enriched CD44v6 CAR-T cells by stress-testing them *in vivo* against high-tumor burdens. Prior to that, in order to identify which NGFR isoform(s) to choose as candidate(s) for further validation, we screened them in a minimal residual-disease setting, i.e., in NSG mice challenged with THP-1 cells 3 days earlier. After infusion, all NGFR isoform-enriched CAR-T cell products could be identified in the peripheral circulation by FACS (Figure 4A). Notably, differences in CAR expression levels observed *in vitro* were confirmed *in vivo*. As expected, only mice treated with CH2CH3-, NWL-, NWS-, or NMS-enriched CD44v6 CAR-T cells, but not those treated with NML-enriched CD44v6 CAR-T cells, benefited from substantial antitumor effects, as indicated by normalization of THP-1 cell-infiltrated liver weight (Figure 4B).

We, therefore, proceeded to test NGFR-enriched CD44v6 CAR-T cells *in vivo* against high-leukemia burdens by excluding those enriched through the NML isoform. To this aim, NSG mice were challenged with THP-1 cells carrying a secreted luciferase that allows the monitoring of tumor growth by simply analyzing blood samples (24). After waiting 14 days for high-leukemia burdens to develop, mice were randomized to receive NWL-, NWS-, or NMS-enriched CAR-T cells. All three NGFR isoform-enriched CD44v6 CAR-T cell products were capable of delaying leukemic progression (Figure 4C) and significantly prolong animal survival (Figure 4D). According with the differences described *in vitro*, NMS-enriched T cells proved the most efficacious, followed by NWL-enriched T cells, which possibly suffered from tonic signaling, and by NWS-enriched T cells, which possibly suffered from low expression levels. In accordance with the high-tumor burden setting, the antitumor effects of all three NGFR isoform-enriched CD44v6 CAR-T cells were accompanied by elevated systemic levels of human IFN-γ, in the absence of IL-6 and IL-10 (Figure 4E). In keeping with higher antitumor efficacy, only mice treated with NMS or NWL-enriched CD44v6 CAR-T cells experienced a transient increase in IL-2 and TNF-α the day after CAR-T cells infusion.

**Figure 5:**
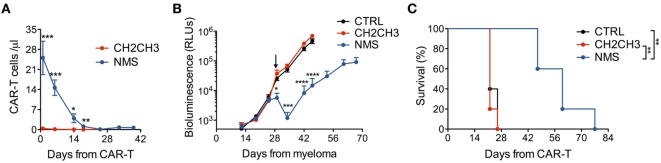
*In vivo* antimyeloma efficacy by nerve growth factor receptor (NGFR)-enriched CD44v6 chimeric antigen receptor (CAR)-T cells. NSG mice were xeno-engrafted with multiple myeloma (MM).1S myeloma cells expressing a secreted luciferase (see Materials and Methods) and, after 28 days (arrow) treated with CD44v6 CAR-T cells enriched through either NGFR mutated short (NMS) (*n* = 5) or CH2CH3 (*n* = 5) or with CTRL, CD19-specific CAR-T cells (*n* = 5). **(A)** Concentration of circulating CD44v6 CAR-T cells over time expressed as number of T cells (identified as hCD3+/hCD45+) per microliter of blood (means ± SEM for the different cohorts). Results from an unpaired *t*-test are indicated when statistically significant (*P ≤ 0.05; **P ≤ 0.01; ***P ≤ 0.001). **(B)** Growth kinetics of MM.1S-luc cells expressed as relative light units (RLUs) (see Materials and Methods, means ± SEM from the different cohorts). Results from an unpaired *t*-test are indicated when statistically significant (*P ≤ 0.05; ***P ≤ 0.001; ****P ≤ 0.0001). **(C)** Survival of the three cohorts over time shown as percentages. Results from Log-rank (Mantel–Cox) tests are indicated when statistically significant (**P < 0.01).

We finally challenged NMS-enriched CD44v6 CAR-T cells against high-myeloma burdens and compared them with CH2CH3-enriched CD44v6 CAR-T cells. In NSG mice randomized for high-tumor burdens by means of the MM.1S-luc technology (see Materials and Methods), NMS-enriched CAR-T cells better engrafted and persisted longer than those enriched through CH2CH3 (Figure 5A), resulting in a superior ability to control myeloma progression (Figure 5B) and to prolong disease-free survival (Figure 5C).

### NGFR-Spaced CAR-T Cells Are Effective Against Multiple Target Antigens

**Figure 6:**
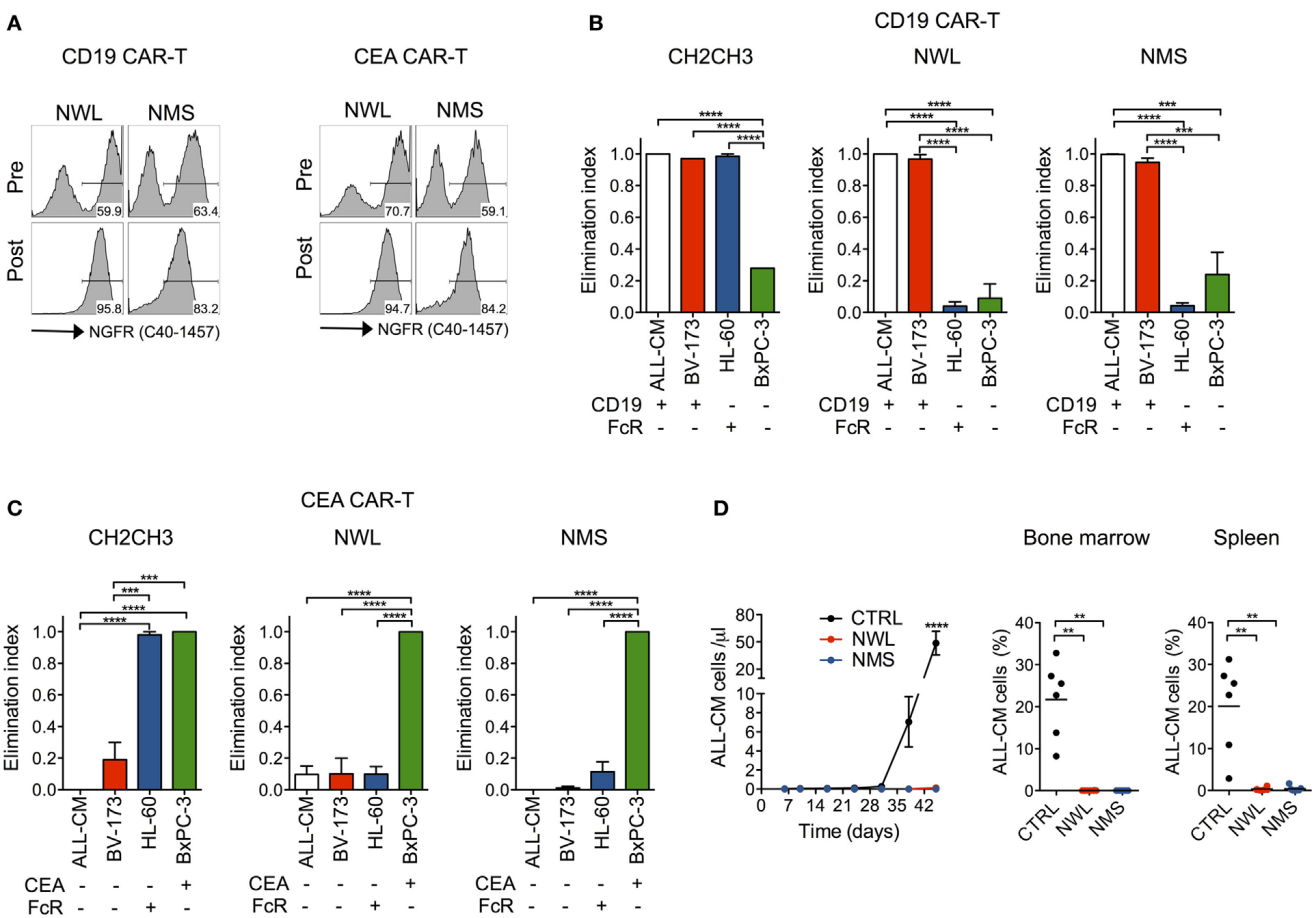
Antitumor efficacy by nerve growth factor receptor (NGFR)-enriched CD19 and CEA chimeric antigen receptor (CAR)-T cells. Primary T cells from healthy donors were stimulated with CD3/CD28-beads, transduced with RVs encoding for CD19 or CEA CARs spaced with NGFR wild-type long (NWL) or NGFR mutated short (NMS), enriched with immuno-magnetic beads and cultured with IL-7/IL-15. **(A)** FACS plots of CAR-T cells from a representative donor (of *n* = 3) are depicted before (pre) and after (post) enrichment. NGFR isoform-enriched CD19 **(B)** or CEA **(C)** CAR-T cells were cocultured at 1:10 E:T ratio with tumor cell lines differentially expressing their respective target antigen and/or Fc receptors (FcRs) (see caption of Figure 1). After 4 days, the elimination of tumor cells by CAR-T cells were analyzed by FACS and expressed as elimination indexes (see Materials and Methods, means ± SEM from *n* = 3 donors). Results from a one-way ANOVA test are indicated when statistically significant (***P ≤ 0.001; ****P ≤ 0.0001). **(D)** NSG mice were xeno-engrafted with ALL-CM leukemic cells and, after three days treated with CD19 CAR-T enriched through either NWL (*n* = 8) or NMS (*n* = 7), or with CTRL (CD44v6-specific) CAR-T cells (*n* = 6). Left: concentration of circulating ALL-CM cells over time expressed as number of T cells (identified as hCD19+/hCD3−) per microliter of blood (means ± SEM for the different cohorts). Right: ALL-CM cell infiltration in the bone marrow and in the spleen is shown for the three cohorts as mean percentages ± SEM. Results from an unpaired *t*-test are indicated when statistically significant (**P ≤ 0.01; ****P ≤ 0.0001).

Desirably, a novel molecular tool aimed at expanding CAR-T cell applications should be functional across multiple target antigens. To address this issue, we generated NWL and NMS-spaced CAR constructs specific for CD19 and CEA. Importantly, after retroviral transduction, both constructs could be visualized by FACS and enabled CAR-T cell enrichment with immuno-magnetic beads (Figure 6A). Moreover, NWL- and NMS-enriched CD19 and CEA CAR-T cells killed CD19+ and CEA+ tumor cells, respectively, while failed to kill cells that did not express the respective target antigen (Figures 6B,C). Similarly to CD44v6, CH2CH3-enriched CD19 and CEA CAR-T cells killed CD19- and CEA- tumor cells expressing FcRs, confirming compromised specificity when using this specific CAR design.

As a proof of concept of the antitumor efficacy of NGFR-spaced CARs against a target antigen different from CD44v6, we xeno-engrafted NSG mice with the CD19+ ALL-CM lymphoid leukemia cells and later infused them with NWL or NMS-enriched CD19 CAR-T cells. In this setting, both NGFR isoform-enriched CD19 CAR-T cell products induced leukemia remissions, as ascertained in peripheral blood and lymphoid organs (Figure 6D).

### NGFR-Spaced CD44v6 CAR T-Cells Can Be Enriched With Clinical-Grade Reagents

**Figure 7:**
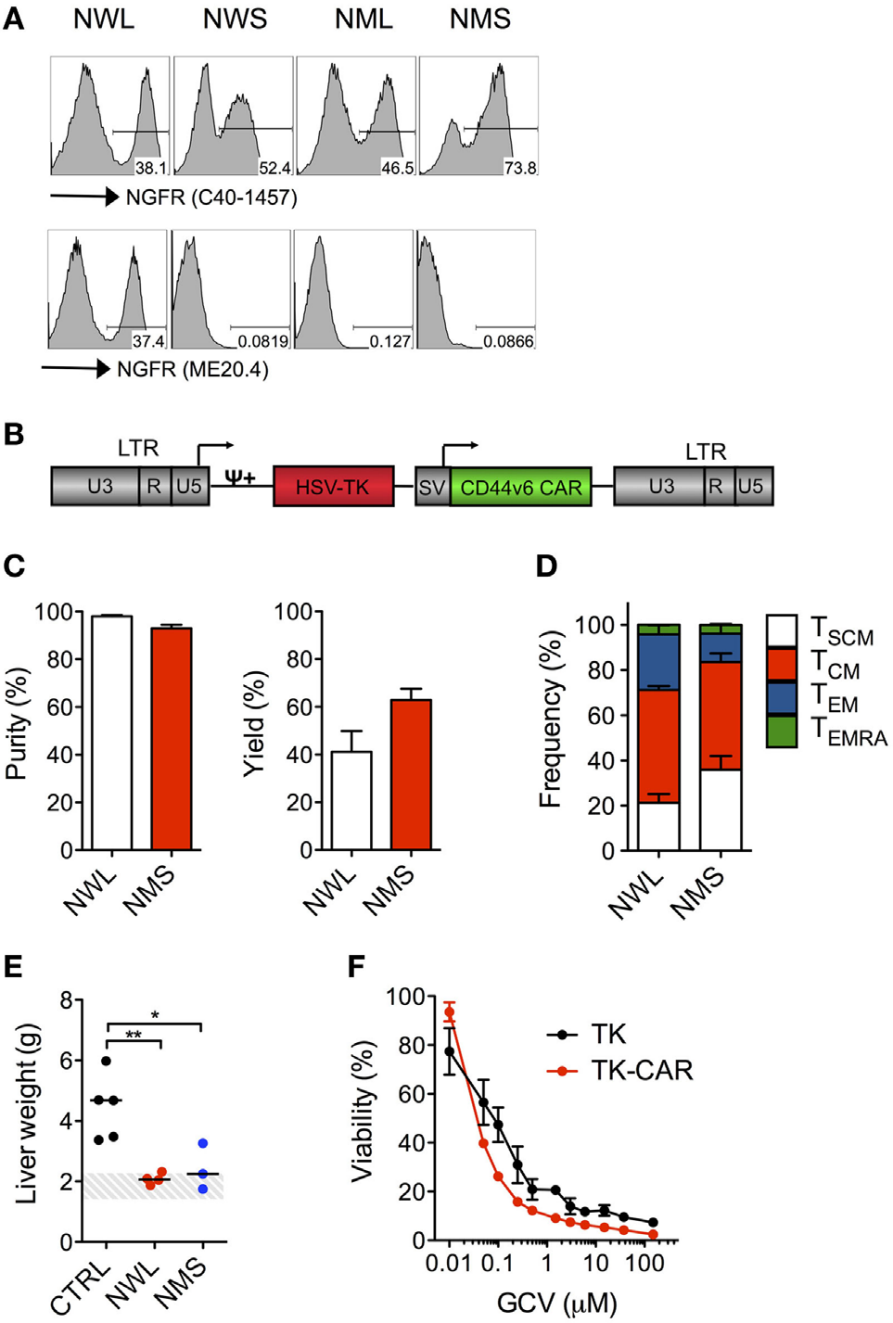
Clinical-grade production of nerve growth factor receptor (NGFR)-spaced CD44v6 chimeric antigen receptor (CAR)-T cells. Primary T cells from healthy donors were stimulated with CD3/CD28-beads and transduced with RVs encoding for CD44v6 CARs spaced with NGFR wild-type long (NWL) or NGFR mutated short (NMS) (see Materials and Methods). **(A)** FACS plots of CAR-T cells from a representative donor (of *n* = 3) stained with either the anti-NGFR monoclonal antibody (mAb) C40-1457 (upper panel) or the anti-NGFR mAb ME20.4 (lower panel). NWL-isoform-spaced CAR T cells were enriched with clinical-grade anti-NGFR immuno-magnetic beads while NMS-isoform-spaced CAR T cells were stained with the PE-conjugated anti-NGFR mAb C40-1457 and enriched with anti-PE immuno-magnetic beads. **(B)** Schematic representation of the retroviral construct expressing the CD44v6 CAR and the thymidine kinase (TK) suicide gene. HSV-TK: Herpes Simplex Virus thymidine kinase suicide gene. CD44v6 CAR: NMS or NWL-isoform-spaced CD44v6 CAR. U3, R, U5: LTR regions. SV: SV40 early promoter. ψ: encapsidation signal. Arrows indicate promoters. **(C)** Purity (left) and yield (right) of CAR-T cells after enrichment (means ± SEM from *n* = 3 donors). Yield was determined by the absolute numbers of NGFR-spaced CAR-T cells in the enriched fraction divided by the absolute numbers of NGFR-spaced CAR-T cells in the starting population. **(D)** Memory phenotypes of NGFR isoform-enriched CD44v6 CAR-T cells analyzed at day 10–13 after bead stimulation (means ± SEM from *n* = 3 donors). TSCM, CD45RA+/CD62L+ stem memory T cells; TCM, CD45RA−/CD62L+ central memory T cells; TEM, CD45RA−/CD62L− effector memory T cells; TEMRA, CD45RA+/CD62L− effector memory RA T cells. **(E)** NSG mice were xeno-engrafted with THP-1 leukemic cells and, after 14 days, treated with CD44v6 CAR-T cells enriched through either NWL (*n* = 4) or NMS (*n* = 3) or with T cells expressing a control, CD19-specific CAR (CTRL, *n* = 5). Forty days later, mice were sacrificed and their liver weighted. THP-1 infiltrated liver weights are shown for the different cohorts (each symbol represents a single mouse and the median value for each group is reported). The dashed area depicts the range of normal liver weight from age/sex-matched normal NSG mice. A representative experiment (of *n* = 3) is shown. Results from a one-way ANOVA test are indicated when statistically significant (*P ≤ 0.05; **P ≤ 0.01). **(F)** Growth suppression of CD44v6 suicidal CAR T cells analyzed after activation with PHA and treatment with increasing concentrations of GCV (means ± SEM from a representative donor). T cells transduced with the original vector expressing the tk suicide gene and the selection marker ΔNGFR were used as comparison.

A NGFR-enriched CD44v6 CAR-T cell product coexpressing the TK suicide gene for switching-off potential toxicities (20, 31) will soon be investigated in a phase I/IIa clinical trial in relapsed/refractory AML and MM (EC-funded H2020 EURE-CART Consortium). During validation, we observed that, while all NGFR isoform-spaced CD44v6 CARs bound the anti-NGFR mAb C40-1457, only the NWL-isoform was able to bind the anti-NGFR mAb ME20.4 (Figure 7A), possibly as a consequence of conformational changes altering epitope accessibility. Since directly conjugated clinical-grade immuno-magnetic beads are based on ME20.4, in addition to NMS we decided to include into further process development also the NWL-isoform. The two NGFR isoform-spaced CD44v6 CARs were cloned along with the TK suicide gene into a clinical-grade bi-cistronic retroviral vector (20), under the control of an internal SV40 promoter, which is far less potent than viral LTRs in driving transgene expression (Figure 7B). After transduction, NWL-spaced suicidal CD44v6 CAR-T cells were enriched with directly conjugated immuno-magnetic beads, while enriching NMS-isoform-spaced suicidal CD44v6 CAR-T cells required a cumbersome two-step procedure, i.e., staining with the PE-conjugated anti-NGFR mAb C40-1457, followed by the use of anti-PE immuno-magnetic beads. In either case, suicidal CD44v6 CAR-T cells were enriched to >90% purity, with yields of 40–60% (Figure 7C). Quite surprisingly, however, at the end of the entire process, the disparities between the two CAR-T cell products were completely abated. Indeed, NWL- and NMS-spaced suicidal CD44v6 CAR T cells displayed a similar differentiation phenotype (Figure 7D), and most importantly, mediated superimposable antitumor effects against high-leukemia burden *in vivo* (Figure 7E), suggesting the attenuation of NWL-induced tonic signaling under the control of the internal promoter SV40. Last but not least, NWL-spaced CD44v6 CAR T cells maintained efficient suicidability upon exposure to GCV, with an IC50 of 0.032 µM compared to 0.059 µM of the original clinical-grade construct (Figure 7F).

## Discussion

Despite widespread and justified excitement, the most recent results of cellular immunotherapy with CAR T cells specific for CD19 (2–8) raise important questions as to long-term antitumor efficacy and safety (36). Moreover, the successful application of CAR-T cells to other hematological malignancies and, possibly, solid tumors remain to be demonstrated, as the choice of additional viable target antigens is so far limited.

If one takes a closer look at the CD19 CAR-T cell products investigated so far in the clinic, beside differences in costimulatory endodomains, stimulation protocols and viral vectors, it is worth noting the significant inter-trial and inter-patient variability in terms of proportion of transduced cells, with average values ranging from 20 (3–5) to 60% (2, 6) in different trials and individual values ranging from 5.5 to 45.3% (5), from 5 to 60% (4), or from 55.1 to 76.8% (6) in different patients. Ideally, to better study the crucial factors influencing the efficacy and safety profiles, CARs would be designed to enable the *in vivo* tracking of receptor-bearing T cells, their phenotypic characterization and possibly their re-isolation for functional *ex vivo* analysis. In addition, the possibility to purify CAR-T cells before infusion into patients would promote the design of standardized protocols for CAR-T cell therapies and facilitate future application in the allogeneic setting. Recently, it has been demonstrated that, though CAR-expressing T cells are expected to exert antitumor effects in the absence of GVHD, the transfer of large numbers of non-transduced T cells is sufficient to increase its incidence and severity (37). A critical role of the costimulatory endodomain has been also highlighted, with 41BB proved more prone to induce lethal GVHD than CD28 (37). Therefore, even if recent clinical studies have reported minimal GVHD incidence in recipients of allogeneic CD19 CAR.28z T cells for the treatment of B-cell malignancies (38), it cannot be excluded a different outcome when changing the treatment schedule, tumor type and CAR construct. In this scenario, having the possibility to purify T cells expressing the CAR and possibly a suicide gene before infusion into patients may drastically increase the safety of the strategy. Recently, introduction of Strep-tag II sequences into specific sites of synthetic CARs and natural TCRs of diverse specificities have been proposed for the easy identification and rapid purification of fully functional engineered T cells (39). In this work, we proposed, as a valuable alternative, the inclusion within the CAR backbone of a novel extracellular spacer based on the low-affinity NGFR. Moreover, we demonstrated the possibility to exploit the NGFR spacer to sort functional CAR-T cells coexpressing the TK suicide gene using clinical-grade vector and reagents, paving the way for a successful exploitation of this strategy in the allogeneic setting.

A truncated version of NGFR, lacking its intracellular signaling domain, has been extensively tested in clinical trials in combination with other therapeutic genes (like the TK suicide gene), proving its suitability as a safe and effective marker for T-cell selection (20, 31). Importantly, we here show that, when inserted within the CAR molecule, NGFR is incapable of signaling in response to soluble NGF, even at manifold supra-physiological concentrations, eliminating concerns about uncontrolled outgrowth of NGFR-enriched CAR-T cells upon potential *in vivo* growth-factor encounter. In addition, when inserted into the CAR molecule, it does not interfere with the suicidal machinery upon coexpression with the TK suicide gene. CARs are modular structures, where all domains may affect CAR-T cell performances (40). Accordingly, the different NGFR isoform-spaced CARs investigated in our study showed functional disparities. For instance, the NWS and the NML design were less expressed than the others, possibly as a consequence of defective protein stability. Accordingly, they showed suboptimal antitumor activity. The NML design in particular failed to show antitumor activity *in vitro*, suggesting defective interaction with the target antigen. On the other hand, the NWL-isoform CAR, which is the one expressed at higher levels and the unique that could be reproducibly enriched with clinical-grade immuno-magnetic beads directly conjugated with an anti-NGFR mAb (clone ME20.4), suffers from tonic signaling, which led to premature T-cell aging and reduced antitumor activity *in vivo*. It was relevant, however, that changing the expression platform and the manufacturing procedure restored the *in vivo* antitumor activity of NWL-isoform CAR-T cells, underlining that multiple variables have to be taken into account when designing a new CAR molecule.

While spacers are required for CAR-T cell targeting of tumor-cell membrane-proximal epitopes (41, 42), the same spacers have been shown to have neutral (27, 40) or even unfavorable (43) effects on the recognition of membrane-distal epitopes. Interestingly, the NGFR extracellular portion has a modular structure, characterized by repeated TNFR cysteine-rich domains, allowing the future evaluation of the possibility to adapt spacer length to different specificities by acting on this modular structure. In this work, for proof-of-concept of the functionality of NGFR-based spacers, we chose as target antigen the CD44 isoform variant 6 (CD44v6), which is knowingly over-expressed in AML (44) and MM (45), as well as on a variety of epithelial tumors (46), and has been implicated in tumor progression and resistance to radio-chemotherapy (47). This choice provides the unique opportunity of tackling multiple tumor indications with a single CAR-T cell product, while simultaneously lowering the probability of immune evasion, as observed with CD19 CAR-T cells (48, 49). We recently developed a CD44v6 CAR and proved its efficacy against AML and MM (25). Regretfully, however, our original CD44v6 CAR included an IgG1-CH2CH3 spacer, which may unduly interact with FcR-expressing myeloid cells, driving off-target activation of CAR-T cells (26). As a consequence, it has been demonstrated that, once infused into NSG mice, CH2CH3-spaced CAR-T cells fail to exert significant antitumor activity because of their premature clearance from the blood stream upon interaction with soluble FcRs (30) or/and trapping in the lung upon interaction with resident phagocytes (27, 29). Interestingly, while we did not observe this detrimental effect of the IgG1-CH2CH3 spacer against low-tumor burdens (25), we clearly observe it against high-tumor burdens, where possibly the number of CAR-T cells available for *in vivo* tumor-cell killing becomes limiting. Several approaches have been proposed to date for abating the off-target effects of CH2CH3 spacers, including their partial or complete removal, or their mutagenization (27, 29, 30). Compared to them, however, while maintaining potent therapeutic effects against high-tumor burdens, the NGFR spacer technology has the advantage of being fully compatible with clinical-grade immuno-magnetic beads. Last but not least, similar results to that obtained with CD44v6 were achieved with two additional target antigens, CD19 and CEA, validating the potential universality of NGFR spacers as safe and effective tools for CAR-T cell enrichment before infusion.

In summary, we have developed and validated a technology enabling the enrichment of CAR-T cells through an NGFR spacer by means of clinical-grade immuno-magnetic beads. A CD44v6 CAR-T cell product implementing this technology and coexpressing the TK suicide gene for switching-off potential toxicities (20, 31) is currently at late stage of process development and will soon be investigated in a phase I/IIa clinical trial in relapsed/refractory AML and MM (EC-funded H2020 EURE-CART Consortium).

## Ethics Statement

This study was carried out in accordance with the recommendations of the “IACUC: Institutional Animal Care and Use Committee (IACUC #468).” The protocol was approved by the “Italian Ministry of Health.”

## Author Contributions

MC designed experiments, performed research, analyzed data and wrote the manuscript. LF, BC, MN, SP, and AS performed research. FC, CT, CLB, and CHB assisted with experimental design and revised the manuscript. AB designed research, analyzed data, wrote the manuscript and acted as senior author of the study.

## Conflict of Interest Statement

CT, SP, AS, and CBordignon are employees of Molmed Spa, whose potential product is studied in this work. FC and CB are consultants to Molmed Spa. AB receives research funding from Molmed Spa.
